# Revolutionizing Epithelial Differentiability Analysis in Small Airway-on-a-Chip Models Using Label-Free Imaging and Computational Techniques

**DOI:** 10.3390/bios14120581

**Published:** 2024-11-29

**Authors:** Shiue-Luen Chen, Ren-Hao Xie, Chong-You Chen, Jia-Wei Yang, Kuan-Yu Hsieh, Xin-Yi Liu, Jia-Yi Xin, Ching-Kai Kung, Johnson H. Y. Chung, Guan-Yu Chen

**Affiliations:** 1 Institute of Biomedical Engineering, College of Electrical and Computer Engineering, National Yang Ming Chiao Tung University, Hsinchu 300093, Taiwan; g199500@gmail.com (S.-L.C.); renhao.ee09@nycu.edu.tw (R.-H.X.); eric-cyc13@nycu.edu.tw (C.-Y.C.); kyhsieh@ibm.com (K.-Y.H.); asd072141@gmail.com (X.-Y.L.); jiayi1029@nycu.edu.tw (J.-Y.X.); kck.ee13@nycu.edu.tw (C.-K.K.); 2 Department of Electronics and Electrical Engineering, College of Electrical and Computer Engineering, National Yang Ming Chiao Tung University, Hsinchu 300093, Taiwan; 3 Anivance AI Corporation, Hsinchu 30010, Taiwan; jack.yang@anivance.io; 4 Graduate Degree Program of College of Electrical and Computer Engineering, National Yang Ming Chiao Tung University, Hsinchu 300093, Taiwan; 5 Intelligent Polymer Research Institute, University of Wollongong, Wollongong, NSW 2500, Australia; jchung6@hotmail.com; 6 Department of Biological Science and Technology, College of Biological Science and Technology, National Yang Ming Chiao Tung University, Hsinchu 300093, Taiwan; 7 Center for Intelligent Drug Systems and Smart Bio-devices (IDS2B), National Yang Ming Chiao Tung University, Hsinchu 300093, Taiwan

**Keywords:** small airway on a chip, deep learning, image recognition, ciliary beating frequency, mucociliary clearance

## Abstract

Organ-on-a-chip (OOC) devices mimic human organs, which can be used for many different applications, including drug development, environmental toxicology, disease models, and physiological assessment. Image data acquisition and analysis from these chips are crucial for advancing research in the field. In this study, we propose a label-free morphology imaging platform compatible with the small airway-on-a-chip system. By integrating deep learning and image recognition techniques, we aim to analyze the differentiability of human small airway epithelial cells (HSAECs). Utilizing cell imaging on day 3 of culture, our approach accurately predicts the differentiability of HSAECs after 4 weeks of incubation. This breakthrough significantly enhances the efficiency and stability of establishing small airway-on-a-chip models. To further enhance our analysis capabilities, we have developed a customized MATLAB program capable of automatically processing ciliated cell beating images and calculating the beating frequency. This program enables continuous monitoring of ciliary beating activity. Additionally, we have introduced an automated fluorescent particle tracking system to evaluate the integrity of mucociliary clearance and validate the accuracy of our deep learning predictions. The integration of deep learning, label-free imaging, and advanced image analysis techniques represents a significant advancement in the fields of drug testing and physiological assessment. This innovative approach offers unprecedented insights into the functioning of the small airway epithelium, empowering researchers with a powerful tool to study respiratory physiology and develop targeted interventions.

## 1. Introduction

Over the past 15 years, the field of organ-on-chip (OoC) systems has rapidly grown as a miniature cell culture platform that replicates the structure and function of human organs [1]. Polydimethylsiloxane (PDMS) has been widely used in fabricating microfluidic chips due to its transparency and compatibility with fluorescence microscopy, allowing for the acquisition of abundant image data [2]. However, there is a need for a streamlined image analysis approach in this field. While 2D/3D cell and tissue cultures have advanced [3,4,5], the analysis of cell states and responses to stimuli is often constrained by the use of stains and biomarkers, limiting measurements to a single time point [6]. Additionally, conventional manual analysis methods are prone to human errors and hinder analytical accuracy [7].

Among the cells in the small airway epithelium, ciliated cells play a crucial role, accounting for 80% of the upper airway epithelium. Ciliary beating frequency (CBF) is typically in the range of 9–20 Hz, varying with factors such as infection, temperature, age, and inflammation [8]. Due to the high oscillatory motion during beating, naked-eye analysis cannot accurately determine CBF. Traditional methods employ sophisticated cinema cameras and high-power microscopes to capture the entire ciliary beat cycle, followed by manual counting to calculate CBF. High-speed digital cameras have replaced film photography and allow for instantaneous analysis, but the counting of individual ciliary beat cycles still relies on manual identification. In recent decades, techniques based on the photoelectric effect and photodiodes have been proposed to enhance the accuracy of CBF analysis [9], but they still require manual recording and calculation, which can be time-consuming and prone to errors.

In addition to CBF analysis, current approaches to assessing epithelial differentiation are primarily based on endpoint staining to confirm the presence of differentiated epithelial cells [5], which often require extended culture periods. This is especially true for HSAEC, where achieving sufficient differentiation can be particularly time-consuming, leading to significant time and cost demands. These limitations restrict the ability to rapidly obtain data and increase overall experimental costs. Moreover, these methods rely on destructive assays, preventing continuous monitoring of cellular differentiation and growth.

**Scheme 1:**
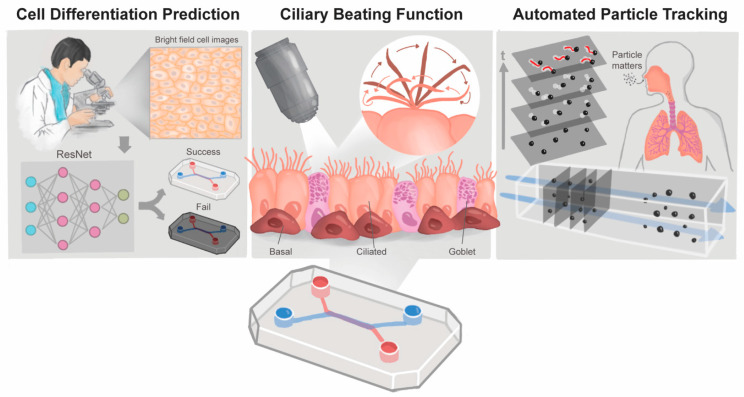
Advanced Image Analysis Methods on Small Airway-on-a-Chip Models. Overview of this study, deep learning was used to predict the differentiation of primary human small airway epithelial cells on an organ-on-a-chip. (**Left**) To observe ciliary beating frequency (CBF) in real time, use MATLAB (R2022a) software development for movie-based automated ciliated cells labeling and CBF calculation. (**Middle**) Finally, under the evaluation of air pollution conditions, the establishment of automated particle tracking technology helps us quickly understand the movement trajectory and directionality of fluorescent particles in the small airway chip (**Right**).

This study proved deep learning and automated image recognition to establish an advanced image analysis pipeline. By utilizing deep learning and image recognition, we enhance the analysis of small airway epithelial cells, providing an improved tool for small airway-on-a-chip image analysis (Scheme 1). For efficient functional analysis of ciliated cells, several automatic image computing models was proposed to track changes in the ciliary beating and mucociliary clearance (MCC) in videos, enabling the calculation of CBF based on the number of frames covered by the peaks of grayscale values and particle tracking. This model can complete the calculation and quantification for each video within 1 min, improving efficiency by 90% compared to manual methods and providing a simpler and faster analysis to assess the physiological function of ciliated cells. Additionally, To predict early cell differentiation and growth success on the chips, an analysis system based on the ResNet [10] deep learning model was developed to assess the survival of small airway epithelium [11,12], significantly reducing experimental time and cost.

By combining automated image recognition, deep learning, and small airway-on-a-chip technology, our study contributes to the advancement of image analysis pipelines in the field. It provides a more effective tool for analyzing the states and functions of small airway epithelial cells, facilitating research and development in the context of small airway-on-a-chip image analysis. The integration of advanced technologies holds great potential for enhancing our understanding of organ function and advancing drug research and development, thereby reducing reliance on animal models in these fields.

## 2. Materials and Methods

### 2.1. Chip Device Manufacturing

The graphic structure of the chip was designed using 3D computer-aided design (CAD) Software-SolidWorks (2019SP3) (Dassault Systemes SA, Vélizy-Villacoublay, France). The device containing two parallel microchannels (top channel, 1000 μm wide × 1000 μm high; bottom channel, 1000 μm wide × 200 μm high; length of channels, 16.7 mm), respectively. After creating a 3D model, the module was prepared with plastic injection molding by BIG BRIGHT Machinery Precision CO., Ltd. (Hsinchu County, Taiwan) for mass manufacturing. The channels were separated by PET membrane (0.4 μm pores) that was purchased from Sterlitech (Auburn, Washington, DC, USA). The membrane was plasma-treated using Plasma Etcher PE-100 (Plasma Etch, Carson City, NV, USA) with 50 watts of oxygen gas for 15 s, sandwiched between the carefully aligned top and bottom channels [13]. This bilayer design enables small airway epithelial cells to inoculate the upper channel with HSAEC and the bottom channel with a culture medium, thus cultivating the air–liquid interface [14].

### 2.2. Small Airway-on-a-Chip Culture

Primary human small airway epithelial cells (HSAECs) were obtained from ATCC. HSAECs were cultured according to the manufacturer’s protocol (PCS-301-010, Information: Male, 16 years, Hispanic/Latino, LOT: 64079184, ATCC) using a small airway epithelial cell growth medium (StemCell, 05040, Cambridge, UK) in T75 plates until 70–80% density. The porous membrane of the device was coated with type-I collagen (A1048301, Thermo Fisher Scientific, Waltham, MA, USA) and stored at 4 °C for 24 h before chip culture. HSAEC were detached via trypsin (SH30042.01, Cytiva, Marlborough, MA, USA) and seeded on the porous membrane coated with type-I collagen in the upper channel of the device at a 5 × 10^6^–8 × 10^6^ cells ml*^−^*^1^. After 3 h, the excess cells were rinsed with a fresh medium and cultured in a submerged state until the cells were fully confluent (usually in 2–3 days). One day after seeding, cells were subjected to dynamic culture. A micropump (#11207801, 10 transfer channels, 2E mechatronic, Kirchheim unter Teck, Germany) was used to circulate at a flow rate of 120 μL min*^−^*^1^. When the cells are confluent, the medium in the upper layer was discarded to form an ALI interface for differentiation. The cells were washed weekly with PBS to remove cell debris, and the related analysis research was conducted on day 33.

### 2.3. Immunofluorescence Staining

The upper and lower channels were washed with DPBS, fixed, and permeabilized with Cytofix/Cytoperm (554722, BD Biosciences, Franklin Lakes, NJ, USA) for 15 min at room temperature and rinsed with DPBS three times. Then, the blocking buffer (1X PBS/5% FBS/1% BSA) was adopted for 60 min at room temperature to minimize the non-specific binding of the proteins. The cells were incubated with primary antibodies for 90 min, washed with DPBS, and incubated with secondary antibodies for 90 min (Table S1). Finally, it was counterstained with DAPI and photographed using a high-content imaging system (Molecular Devices, San Jose, CA, USA) and confocal microscopy (Leica, Wetzlar, Germany).

### 2.4. Image-Based Prediction of Cell Differentiation

#### 2.4.1. HSAEC Dataset

Images of the small airway epithelial cells were photographed on day 3 in the microchannel, and then immunofluorescence staining was performed on day 31–38 of culture. The areas with ZO-1 confirmed by staining were determined as “differentiable tissue”, and the areas without ZO-1 were determined as “non-differentiable tissue”. The training dataset was created by labeled 224 × 224 pixel from day 3 cell images. On the same time, the training dataset consists of images of two categories: differentiable tissue and non-differentiable tissue, which were labeled as “0” and “1”, respectively.

#### 2.4.2. Data Augmentation

Data augmentation was performed by flipping and rotating the images by 90°, 180°, and 270°, and adding Gaussian noise.

#### 2.4.3. Establishment and Validation of the Small Airway Survival Recognition Model

Four common pre-trained convolutional neural network (CNN) models were implemented to classify differentiable tissue versus non-differentiable tissue, including AlexNet [15], VGG-16 [16], GoogLeNet [17], and ResNet. 5-fold cross-validation (CV) was used to validate the accuracy of the model. The dataset was split into five equal parts for each training, 80% of the split dataset were randomly selected as the training set, and the rest were used for validation. The model was trained five times, and accuracy was calculated for each training. The 5-fold cross-validation accuracy was yielded by averaging the mean of accuracy from each training.

#### 2.4.4. Feature Visualization

To visualize the acquired feature area, ScoreCAM [18] was. This method utilized the Softmax score as a weight and eliminated the dependence on unstable gradients, by which the featured area could be presented as a heat map.

### 2.5. The Automated Ciliary Beating Frequency Calculating Model

To observe the ciliary beating, the system comprised a high-speed camera (SP 150, Sage Vision Co., Ltd., New Taipei City, Taiwan), USB3.0 port, dedicated software (SG View, version 4.11, Sage Vision Co., Ltd., New Taipei City, Taiwan), and an inverted microscope (Eclipse Ti2-U, Nikon, Tokyo, Japan). The contrast objective lens employed was 20×/Ph1. With an exposure time and rate of 10 ms and 100 frames per second (fps), respectively, three to five randomly selected different fields of view were obtained to record the ciliary beating for 10 s, and the acquired resolution was 1440 × 1080. The recording was imported into MATLAB for analysis, and the total image read out was 990–1000 frames. First, the 2nd–100th frames were extracted. The grayscale value of each frame was subtracted from the grayscale value of the 1st frame, and each frame derived a relative change from the 1st frame. By superimposing and averaging the number of changes in each frame, a corresponding coordinate diagram with the number of changes was obtained. Then, each point was added and averaged to obtain a value, which was set as the threshold. The coordinate positions lower than this threshold value were eliminated, and the remaining coordinate positions were the ciliary beating areas. Next, the grayscale values of each image and for each read position were read, and several peaks were captured within the calculation time, which was then divided by the recording time to obtain the ciliary beat frequency. After the ciliary beat frequency of each point was obtained, the pixels at the corresponding position were presented in a heat map, and the percentage of the total number of cilia in the visual field corresponding to the frequency distribution was calculated to facilitate the subsequent quantitative analysis.

### 2.6. Fluorescent Particles Tracking Analysis

To analyze the MCC capacity of the small airway epithelium, the chips from the day 30–35 of differentiation were selected and passed through a trans-aerosol generator (Aeroneb*^®^* Lab, ANP-1100). In addition, 2-µm FluoSpheres™ (carboxylate-modified microspheres, 2-μm red fluorescence (580/605), 2% solids, F8826, Thermo Fisher Scientific) fluorescent particles were exposed to the upper layer of the chips. After the fluorescent particles adhered to the mucus, the exposure time was set as 10 ms and 30 fps using a CCD (DS-Qi2, Nikon, Japan), and randomly recorded three to five fields of view, such that the recording time was 10–60 s, and the acquired resolution was 536 × 536. The recording was analyzed in MATLAB, first by acquiring the first frame, and then the threshold was set to filter out the excess noise on the screen. Subsequently, the centroid position of the fluorescence point was acquired, and the moving position of the centroid in the next frame of the recording was determined to superimpose and draw the particle motion trajectory. However, the particles might adhere to the cilia, and the calculation might be miscalculated. Hence, all the trajectories were ultimately added and averaged, trajectories with a difference of 70% were eliminated, and trajectories that remained on the cilia were eliminated. Finally, the trajectory map of time versus motion and the rose diagram for determining the directionality were generated.

### 2.7. Statistical Analysis

GraphPad Prism 8.0 (GraphPad software) was used to analyze the data and plotted graphs. The data were expressed as the mean ± the standard error of the mean. The statistical differences were analyzed using the one-way ANOVA and *p* values less than 0.05 were considered statistically significant. * *p* < 0.05; ** *p* < 0.01; *** *p* < 0.001; ns, non-significant.

## 3. Results and Discussion

### 3.1. Mass Production of Polycarbonate(PC) Chips and Small Airway-on-a-Chip Differentiation

**Figure 1:**
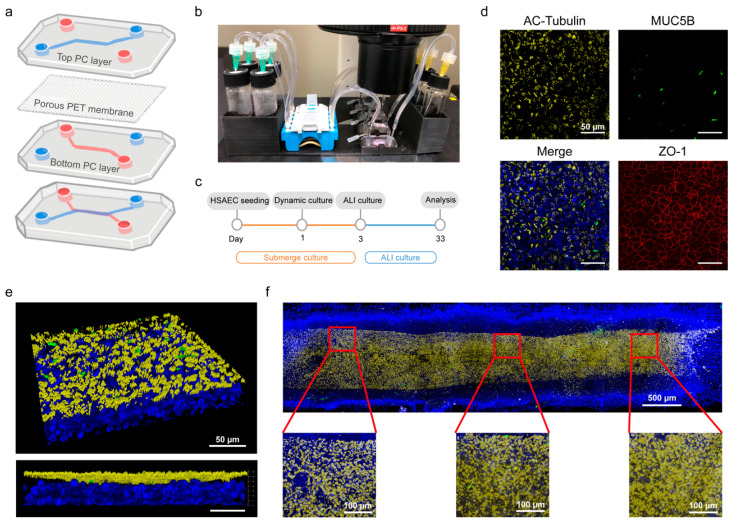
Establish and analyze the small airway epithelium differentiation model. (**a**) Schematic diagram of small airway-on-chip. The chip is a two-channel microfluidic cell culture device composed of air and medium channels. (**b**) Illustration of chip holder and cell image observation platform. (**c**) Timeline of HSAEC differentiation. (**d**) The confocal images show the ciliated, goblet cells and barrier function distribution. Scale bar = 50 μm. (**e**) 3D confocal image of a small airway-on-a-chip model. Scale bar = 50 μm. (**f**) High content image of a high resolution (20*×*) scan. Cells were stained with DAPI (blue), Ac-tubulin (yellow) and MUC5B (green). Scale bar = 500 μm and 100 μm.

The multilayered PC devices contain two parallel channels divided by a porous PET membrane is shown in Figure 1a. Plastic injection modeling was used to fabricate the PC. In this manner, approximately 1000 PC chips can be fabricated per hour [13]. The global OoC market is estimated to be growing at a compound annual growth rate of 30% [19]. Plastic injection molding can reduce the cost of chips (approximately 3–5 USD a piece) to improve the economic benefits of the mass market. In addition, this chip fabrication process is considerably time-saving compared to PDMS cast molding.

To acquire high quality image data from our chips for training a deep learning model to predict the HSAEC differentiation, we designed and 3D-printed a holder to mount four chips simultaneously for stable microscopic photography. The holder contained grooves to place medium glass vials, a platform for stapling a micropump, and holding the chips for image acquisition. A hollow design underneath the chip allows the system to be operated under a microscope for cell image acquisition (Figure 1b). The medium in the chip was cycled by micropump at a flow rate of 120 μL min*^−^*^1^. The PET porous membrane is coated with extracellular matrix with collagen-I coating the day before the cell seeding. One day after seeding the cells, dynamic culture began and the medium in the upper layer was removed on day 3 to form ALI culture for differentiation. The small airway epithelial cells differentiated in a biomimetic human environment and analyzed on day 33 (Figure 1c). Based on our previous studies [20], complete stabilization of cell differentiation typically occurs between 3 and 5 weeks of culture, supporting our selection of day 33 as an appropriate time point for analysis. Furthermore, our observations confirmed that the differentiation state was most stable around the fourth week, aligning with our previous findings. According to literature [21], the thickness of the epithelial cell layer gradually increases to 30–40 µm and then is relatively constant. In our previous work, the number of ciliated cells gradually reached a peak during the day 24 to 31 and was relatively stable afterward, with a wide range of cell thickening [20]. Basal cells are considered progenitor cells that are able to differentiate into three different morphologies. We stained and analyzed the differentiated epithelial cells on day 33. Based on previous work, we validated the functional cell types of small airway epithelium differentiated by this method, with the respective distribution of basal, ciliated, and goblet cells at 7.3 ± 3%, 45 ± 1%, and 8.4 ± 1%, consistent with in vivo data in humans [20]. Therefore, in this study, we confirmed the establishment of well-differentiated small airway epithelial cells through the tight junction, featuring zonula occludens-1 (ZO-1), characteristic epithelial pebble morphology, goblet cells, and dense ciliated cell cover (Figure 1d and Figure S1). The 3D confocal images showed that the thickness of the entire cell layer was approximately 30–40 μm. The ciliated cells distribute at approximately 10 μm at the top of the cell layer, with cilia length of ~10–15 μm. They were the main functional cells of the small airway epithelium and assisted in removing foreign bodies in epithelial cells through a regular ciliary beat (Figure 1e). To quantify the ratio of ciliated cells to mucous cells, we observed the distribution of cell differentiation within the complete flow channel using the stitch function of the high-content image system (Figure 1f).

### 3.2. Predicting Differentiation Outcomes in Small Airway Epithelial Cells Using Deep Learning

**Figure 2:**
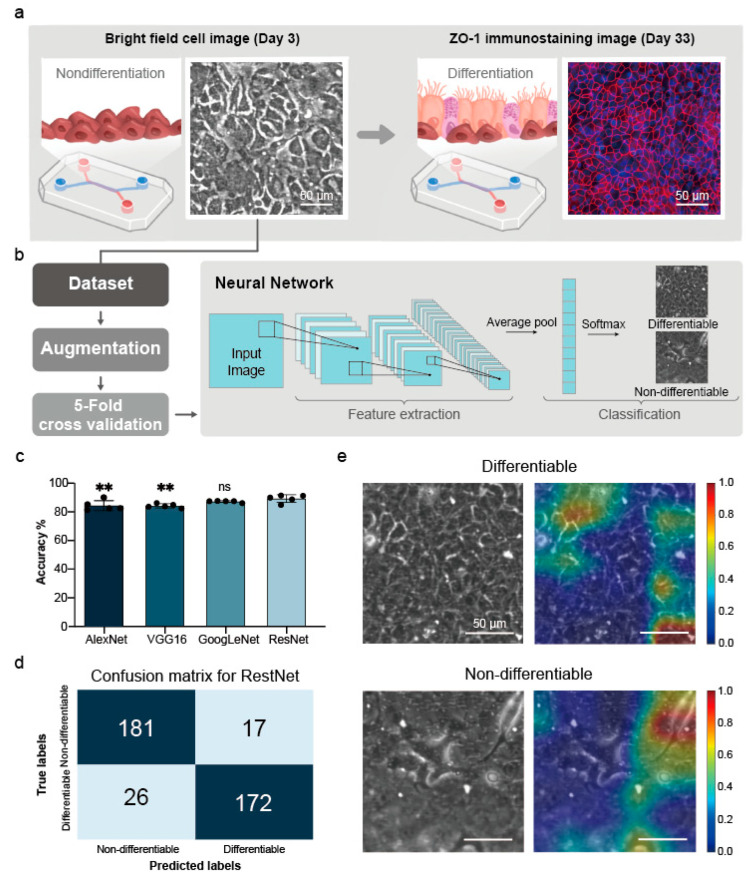
Establish and analyze the small airway epithelium infection differentiation model. (**a**) Use the day 3 bright-field images to create a dataset and use the ZO-1 performance of the day 33 in the same area as the label standard. Scale bar = 50 μm. (**b**) The established dataset is augmented by data, and then 5-fold cross-validation is used to verify the CNN model to distinguish success tissue or fail tissue. (**c**) Prediction results of four classic CNN models. Data are shown as the mean  ±  SD, and one-way ANOVA determines statistical significance. ns: not significant; ** *p* < 0.01. (**d**) Overall confusion matrix generated by ResNet after 50 epochs of training, the horizontal axis refers to the grade predicted from the model, and the vertical axis is based on the marker of ZO-1. The blue brightness is proportional to the value of each cell in the matrix. (**e**) ScoreCAM highlights the grabbed feature regions, blue for low attention and red for close attention. Scale bar = 50 μm.

To improve the efficacy in long-term culture of microfluidic small airway epithelial cells, we used a deep learning image-based approach to create a model for small airway epithelial tissue differentiation. We captured the entire microfluidic channel of cells under bright-field imaging on day 3 with a size of 1608 × 1608. Then, we segmented the images into 224 × 224 images (Figure 2a) to establish a dataset for labeling with the fluorescence staining image of the same region on day 33 (Figure 2b). According to previous studies [20], cells are typically transferred to ALI culture on day 3, and we have observed that differentiation under ALI conditions is strongly influenced by the initial submerge culture phase. Therefore, we chose day 3, prior to the ALI transition, to capture cell images that could serve as inputs for predicting differentiation outcomes. Finally, we calculated the overall accuracy of the cell differentiation efficiency in the microfluidic channel reached 89%.

#### 3.2.1. Dataset Results

The differentiation of small airway is dominated by basal cells [22]. Therefore, it is important to determine whether cells can be differentiated efficiently at the initial stage. In addition, the compactness of ZO-1 of small airway is critical for differentiation [23]. For ZO-1, we identified the differentiable tissue and non-differentiable tissue areas of small airway on the day 31 to 38 of culture and associated the circled areas with the areas on the day 3 (Figure S2).

#### 3.2.2. Data Augmentation Results

We employed the data augmentation technology [24] to prevent over-fitting originating from the insufficiency of training data. Cell culture in microfluidic devices simulates human physiological tissues through dynamic culture in a microenvironment. Compared to conventional culture environments (e.g., well plates or Transwell), microfluidic cell culture has a higher complexity, thus the number of data that could be generated per chip is fewer than the previous methods. Data augmentation has been proven as an effective approach to help increase the amount of data from existing data, thus improving the model accuracy [25]. Therefore, we created new tissue images of small airway epithelial cells for the training set through data augmentation. Usually, virtual data can be created through cropping, flipping, rotation, and noise addition. In this study, we used different rotation angles (including 90°, 270°, and 360°) and Gaussian Blur to create new images. Figure S3 shows the created small airway image. The augmented dataset consists of 1584 images.

#### 3.2.3. Five-Fold Cross-Validation Results

To evaluate the performances of different models (e.g., AlexNet, VGG16, GooLeNet, and ResNet) in using our dataset, the mean accuracy obtained by five-fold cross-validation were 84.09% ± 3.54%, 84.09% ± 1.43%, 87.12% ± 0.59%, and 89.14% ± 2.65% (Figure 2c and Table S2). The ResNet model is the best among these models, this may be due to its largest convolutional depth, resulting in the highest accuracy. The convolution depth of GoogLeNet, AlexNet and VGG16 are less deep, so the performance is not as good as ResNet. To evaluate whether the ResNet model could converge correctly, we extracted the accuracy of training and validation of a single fold (Figure S4a) and the accuracy increased quickly. The training accuracy was approximately 85% at epoch 10, while the validation accuracy was approximately 100% after approximately 3 epochs. In addition, we analyzed the classification results based on the results of this fold and classified its distribution in Figure S4b through a confusion matrix. Most classification results were correct and consistent with the trend indicated in Figure S4a. All validation set results were aggregated into a confusion matrix (Figure 2d). For both differentiable tissue and non-differentiable tissue, the accuracy was approximately 89%. Previously, the prediction of differentiation in most cases of cell culture was based on the differentiation of a single cell [26,27].

#### 3.2.4. Feature Visualization Results

Finally, we used ScoreCAM [18] to investigate what features are captured by the deep learning model to identify the success of tissue culture. This method can quickly capture the trained features (Figure 2e). We found that ScoreCAM focuses on areas where there are clear barriers to differentiable tissue, whereas, in non-differentiable tissue, it focuses on areas that appear to be fibrotic. While the current automated analysis method is confined to predicting the differentiation of HSAECs, its benefits extend beyond this specific context. Not only does it facilitate stable tissue culture in microfluidic systems through integrating deep learning, but it also effectively reduces unnecessary expenses. Furthermore, using deep learning techniques can significantly lower the entry threshold for incorporating small airway epithelial cell culture, enabling more researchers to contribute to this field and catalyze advancements in respiratory-related research.

Our findings align with existing literature regarding the crucial role of tight junctions in cellular differentiation. Tight junctions play a vital role in maintaining epithelial tissues’ integrity and barrier function [28]. Previous studies have demonstrated that the absence of tight junctions hampers the differentiation process, impeding the formation of mature tissue structures. Consistent with these findings, our results indicate that the deep learning model, via ScoreCAM, emphasizes the importance of tight junctions in accurately predicting successful tissue culture. By harnessing the capabilities of deep learning algorithms, we can automate tissue culture analysis, leading to enhanced accuracy and efficiency. Tributes to advancing microfluidic technologies also hold broader implications for various research areas.

### 3.3. Analysis of Ciliary Beat Images

**Figure 3:**
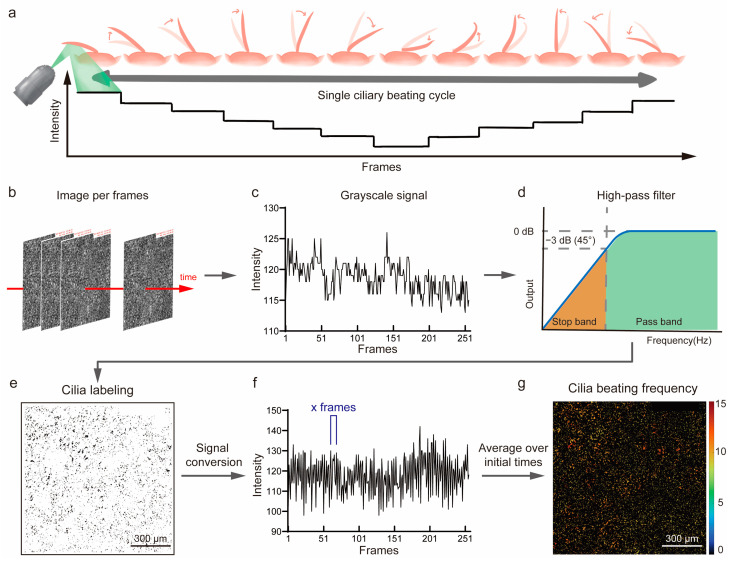
The flow chart of the ciliary beating frequency analysis system. (**a**) Schematic diagram of ciliary beating corresponding to changes in light intensity. (**b**) Input each frame of ciliary beating video. (**c**) Detect light intensity changes and convert to grayscale signals. (**d**) Filter the noise with a high-pass filter. (**e**) Obtain ciliary beating positions and labeling. Scale bar = 300 μm. (**f**) Record the number of light intensity changes at each position. (**g**) The output of ciliary beating area’s distribution corresponds to the beating frequency. Scale bar = 300 μm.

In the preceding section, we microscopically confirmed the differentiation of cilia in OoC and successfully predicted the area of HSAEC differentiation using a deep learning model. Ciliated cells provide an important physical barrier in the lung [29]. Each cilium has a cycle motion of the effective and recovery stroke movement [30]. In our previous work [20], CBF measurement was performed by counting the ciliary beat of an image file visually. Although this method could provide an accurate measurement, it is time-consuming and not capable for real-time analysis. Current CBF measuring techniques are mainly achieved by calculating the light intensity change in the ciliary beat using a high-speed camera to record images. The capture rate of a high-speed camera is usually 85 to 500 fps. Fast Fourier transform (FFT) is considered the standard for CBF estimation. However, the FFT may cause multiple harmonics, which may cause incorrect results of CBF calculation [31]. The ciliary movement in the respiratory tract uses isochronous waves to propel the mucus layer (Figure 3a). To verify whether the differentiated ciliated cells predicted by the deep learning model have normal physiological functions, we verified the functionality of ciliated cells by measuring their CBF, which is a common indicator to measure the functions of ciliated cells. Here, we proposed an automated CBF measurement approach by using a MATLAB program to read the images (Figure 3b) and capture the first 100 frames to calculate the light intensity change in each frame against the first frame. Then we treated each pixel point as a cilium and used light intensity change to calculate the average light intensity change in each pixel point (Figure 3c). Finally, we summated the light intensity changes in all pixel points and used a high pass filter to reduce the signal interferences (Figure 3d) to determine the area of the ciliary beat (Figure 3e). This calculation method enabled quick capturing of locations from a small amount of data, and thus new calculations could simply focus on the captured area. We then read the light intensity change in the captured area and calculated the number of peaks appearing within a certain time (Figure 3f), thus determining the CBF of the area. Compared to previous methods in which all positions are captured excluding unwanted positions, this method enables us to quickly capture positions and complete the calculation. Subsequently, we applied the beat areas to a frequency heat map, allowing us to quickly evaluate the beat status of each area. Finally, we visually compared the ciliary beat status between our calculation method and eye 0.2× slow play calculation. The results showed that the two methods were statistically consistent (Figure S5). Secondly, we compared the computer and eyes calculations of CBF across a larger field of view. The results demonstrated that the computer provided a more objective measurement of the overall CBF (Figure 3g). To observe the frequency distribution of CBF the calculation results were plotted as a histogram (Figure S6), enabling us to quickly verify whether the overall CBF was concentrated on a certain frequency or was inconsistent.

### 3.4. Automated Fluorescent Particle Tracking

**Figure 4:**
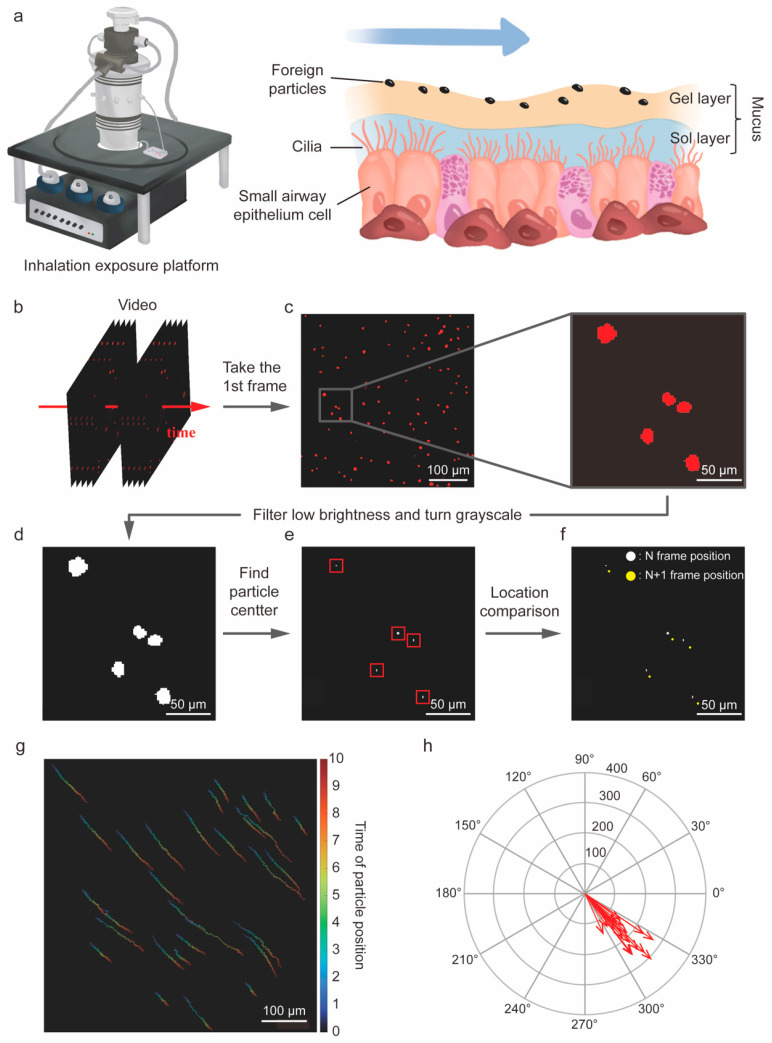
Establishment of fluorescent particle tracking system. (**a**) Schematic diagram of mucociliary clearance observed by fluorescent particles adhering to the mucus layer. (**b**) Input the fluorescent particle video and preprocess each frame. (**c**) Take the first frame to find out the fluorescent particles position. Scale bar = 100, 50 μm. (**d**) Filter low brightness and turn grayscale. Scale bar = 50 μm. (**e**) The system can automatically locate the particle center. Scale bar = 50 μm. (**f**) Detect the position of the next frame. Scale bar = 50 μm. (**g**) The output of particle trajectories. Trace color changes over time. Scale bar = 100 μm. (**h**) Using a rose diagram represents the particles’ moving direction and moving distance.

Small airway-on-a-chip models are widely used to evaluate the damage caused by airborne particles and efficiency of drug delivery. Usually, they entail collection, generation, and analysis of numerous images. In contrast, traditional methods are based on manual calculations by slowing the fluorescence of microscopic images. They provide accurate results but are very time-consuming. MCC is an important tissue function in small airway epithelial tissues [32]. It is a clearing mechanism that removes foreign substances mainly by pushing the upper-layer mucus through the ciliary beat. A directional mucus movement can be observed when the cilium density is high [33]. To observe the mucus movement rate in the MCC, we exposed fluorescent particles to small airway epithelial from day 30 to 35 through an atomization generator in OoC for five minutes and observed mucus movement velocity by tracking the fluorescent particles (Figure 4a). We then used a charge coupled device (CCD) to record the movement and trajectory of fluorescent particles cleared by cilia (Video S1). To analyze the clearance status, we developed an automated particle tracking system using MATLAB and recorded the fluorescent particle removal videos and input them to the analysis system to process each frame (Figure 4b). After reading the first frame (Figure 4c), a threshold was set to filter out the excess fluorescence noise and converted to grayscale (Figure 4d) to prevent circling any coordinates that did not indicate fluorescent particle positions. The pixel points of fluorescent particles were very small. To prevent subsequent particle tracking errors, we initially captured the center-of-mass position of a fluorescent particle (Figure 4e), and then selected eight surrounding pixel positions. Compared to the manual capture and software-based analysis of object positions, we could automatically capture object positions and analyze their movement trajectory more quickly and effectively (Figure 4f). From the results, we determined that if fluorescent particles in an image adhered to cilia and beat, they were also judged to be moving. Therefore, we removed the trajectories that failed to reach 50% of the average amount of movement, thus generating a complete movement trajectory chart (Figure 4g). To interpret the directionality of particle movement more quickly, we used the rose diagram to plot the moving distance and direction of the trajectory to check whether the overall directionality was consistent (Figure 4h). Conventionally, the fluorescent particle movement of an in vitro model was mainly observed visually or by initially manually circling some fluorescent particles in an image, and then calculating their trajectory through specific software [34]. The existing commercially available software does not exclude the ingoing or outgoing fluorescent particles, resulting in a possible underestimation of the MCC capacity. Here, we addressed these limitations by using an automated particle tracking program. The analysis showed that the cultivated HSAEC tissues could effectively generate the MCC. Moreover, the directionality analysis revealed the consistency of movement direction.

## 4. Conclusions

The utilization of microfluidic technology in OoC offers an enormous advantage by simulating the physiological microenvironment and creating a long-lasting, stable 2D/3D environment for cell culture, providing researchers with a valuable platform for exploring cell growth, drug screening and disease tracking. OoC not only serves as a powerful tool for studying human biology but also provides an innovative alternative animal platform for drug development. By accurately mimicking the functionality and responses of human organs, organs-on-chips offer a more ethical and efficient approach to evaluating the safety and efficacy of potential drugs.

In our research, we have also focused on addressing specific challenges in the field. Traditional immunostaining methods, which can only provide one-time results, have been limited by an inefficient data analysis pipeline. To overcome this, we have introduced multiple image analysis techniques for examining cell conditions on the small airway-on-a-chip model. Our approach includes the development of a deep learning model that utilizes bright-field cell images collected on day 3 of the initial stage of culture to predict cell differentiation efficiency. Remarkably, our model achieved an impressive accuracy of 89% in predicting the area of small airway epithelium differentiation.

Furthermore, we have developed a CBF video analysis system using a customized MATLAB program. This system automates the preprocessing of ciliary beating videos and calculates CBF. By enabling the efficient calculation of small-area CBF and generating frequency distribution histograms in minutes, our system significantly enhances data analysis and provides real-time results without disrupting the ongoing experiment. This advancement not only saves time but also improves the overall efficiency of studying ciliary cells and their responses.

Moreover, our research has led to the automation of fluorescent particle tracking, which has the potential to revolutionize inhaled drug deposition testing. By automatically tracking the pathway, directionality, and removal speed of fluorescent particles, we can gain insights into the MCC ability and evaluate the effectiveness of inhaled drugs. This development has implications for optimizing drug delivery mechanisms and enhancing respiratory treatments.

In conclusion, our work shows the potential of image-based deep learning in predicting cell differentiation and developing a scalable, automatic image analysis pipeline. By providing a customizable and end-to-end platform for studying various cell types, we can significantly reduce analysis time and accelerate the production of highly complex systems in the fields of medical and tissue engineering. Additionally, the innovative use of organs-on-chips as an alternative animal platform for drug development and the development of particle tracking systems for inhaled drug deposition testing further expand the applications and impact of this groundbreaking technology.

## Data Availability

Data will be provided upon request from the corresponding author.

## Supplementary Materials

The following supporting information can be downloaded at: https://www.mdpi.com/article/10.3390/bios14120581/s1, Figure S1: Quantification of Small Airway Epithelial Differentiation; Figure S2: Dataset for deep learning model training; Figure S3: Effects of the four augmentation methods applied to HSAEC image; Figure S4: The accuracy of the ResNet model in one data fold; Figure S5: CBF calculating between computational model and eyes; Figure S6: Calculate the frequency distribution of CBF on the screen; Table S1: Summary of antibodies used in this study; Video S1: Fluorescent particles cleared by cilia.
