# Supplementary material for: Revolutionizing Epithelial Differentiability Analysis in Small Airway-on-a-Chip Models Using Label-Free Imaging and Computational Techniques
Source: Biosensors (Basel). 2024 Nov 29;14(12):581. doi: 10.3390/bios14120581 (PMC11675036; doi:10.3390/bios14120581)
Supplement: Supplementary file 1 [file biosensors-14-00581-s001.zip › biosensors-3269927-supplementary.pdf]

Supplementary Information

Revolutionizing Epithelial Differentiability Analysis in Small Airway-on-a-Chip Models Using Label-Free Imaging and Computational Techniques

Shiue-Luen Chen [1,2,†], Ren-Hao Xie [1,2,†], Chong-You Chen[1,2], Jia-Wei Yang[3], Kuan Yu Hsieh[4], Xin-Yi Liu[1], Jia-Yi Xin[1], Ching-Kai Kung[5], Johnson H.Y. Chung[6] and Guan-Yu Chen [1,2,7,8,*]

1 Institute of Biomedical Engineering, College of Electrical and Computer Engineering, National Yang Ming Chiao Tung University, Hsinchu, 300093, Taiwan
2 Department of Electronics and Electrical Engineering, College of Electrical and Computer Engineering, National Yang Ming Chiao Tung University, Hsinchu, 300093, Taiwan
3 Anivance AI Corporation, Hsinchu, Taiwan
4 IBM T.J. Watson Research Center, 1101 Kitchawan Road, Yorktown Heights, Yorktown, NY, 10598 USA
5 Graduate Degree Program of College of Electrical and Computer Engineering, National Yang Ming Chiao Tung University, Hsinchu, 300093, Taiwan
6 Intelligent Polymer Research Institute, Institute for Innovative Materials, University of Wollongong, 2500, NSW, Australia
7 Department of Biological Science and Technology, College of Biological Science and Technology, National Yang Ming Chiao Tung University, Hsinchu, Taiwan
8 Center for Intelligent Drug Systems and Smart Bio-devices (IDS2B), National Yang Ming Chiao Tung University, Hsinchu, Taiwan
* Correspondence: guanyu@nycu.edu.tw; Tel.: +886-3-573-1920
† These authors contributed equally to this work.

Contents

Figure S1. Quantification of small airway epithelial differentiation.
Figure S2. Dataset for deep learning model training.
Figure S3. Effects of the four augmentation methods applied to HSAEC image.
Figure S4. The accuracy of the ResNet model in one data fold.
Figure S5. CBF calculating between computational model and eyes.
Figure S6. Calculate the frequency distribution of CBF on the screen.
Table S1. Summary of antibodies used in this study.
Video S1. Fluorescent particles cleared by cilia.

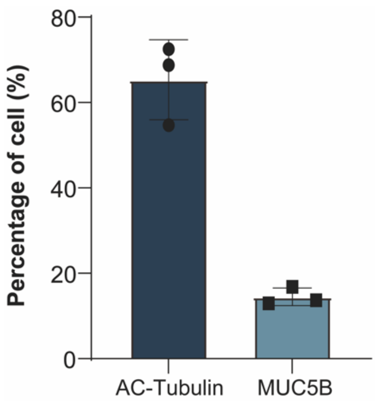

Figure S1. Quantification of small airway epithelial differentiation. The quantitative of ciliated and goblet cell expression.

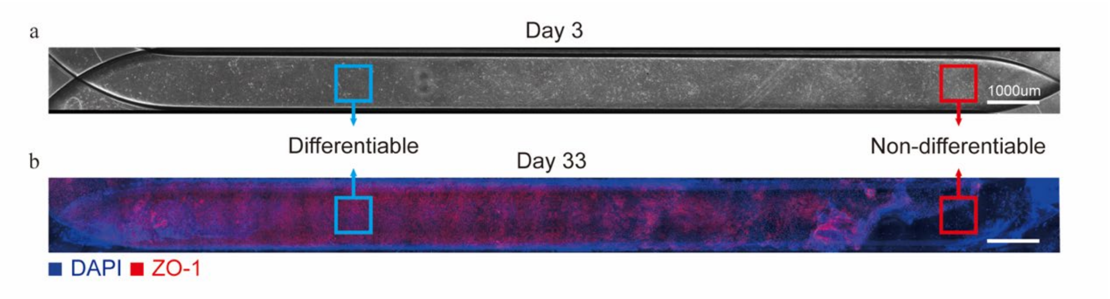

Figure S2. Dataset for deep learning model training. (a) Day 3 bright field image of the full flow channel. (b) Day 33 fluorescent staining image of the full flow channel. Scale bar = 1000 μm.

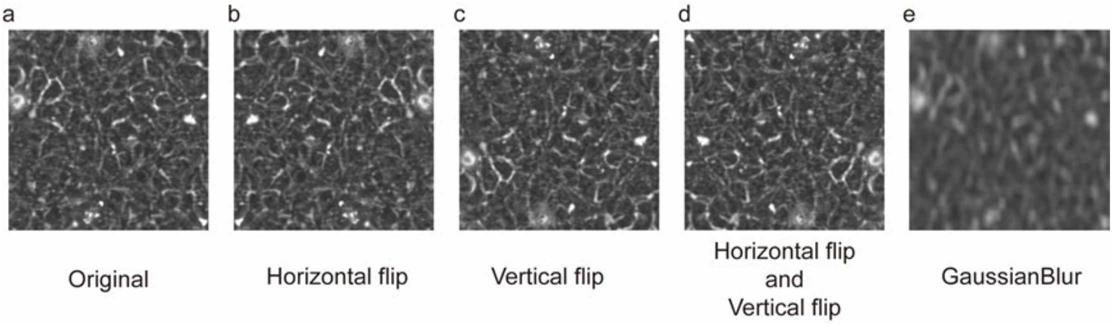

Figure S3. Effects of the four augmentation methods applied to HSAEC image. (a) Original image. (b) Horizontal flip. (c) Vertical flip. (d) Horizontal flip first, then vertical flip. (e) Gaussian blur.

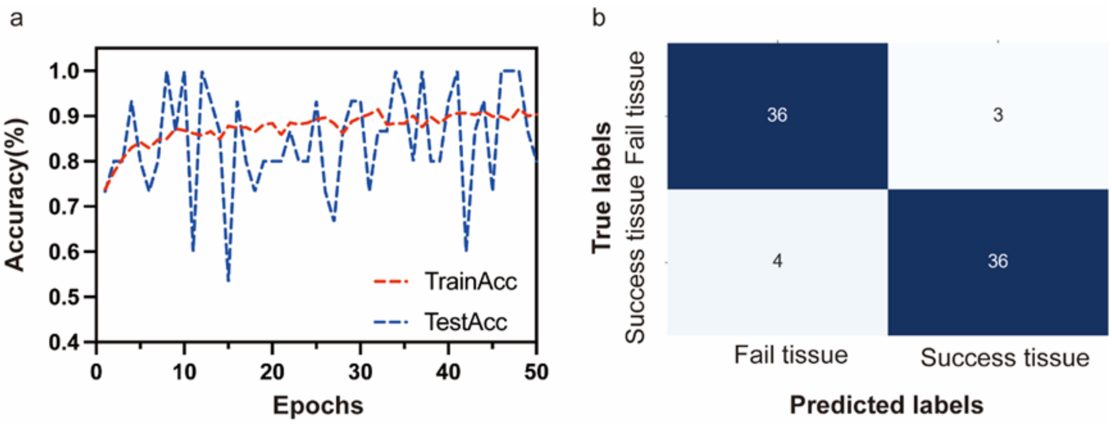

Figure S4. The accuracy of the ResNet model in one data fold. (a) Train and validation accuracy plots during ResNet training for one data fold. (b) Confusion matrix generated by the ResNet after 50 epochs of training and the histology predictions for one data fold. The blue brightness is proportional to the value of each cell in the matrix.

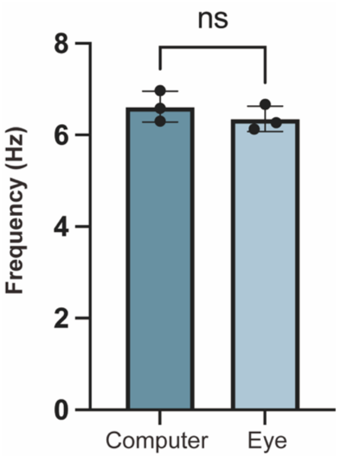

Figure S5. CBF calculating between computational model and eyes. Comparing computer-based calculation of cilia beating frequency with the calculation of ciliary beats by using the eye at 0.2x speed. ns: not significant.

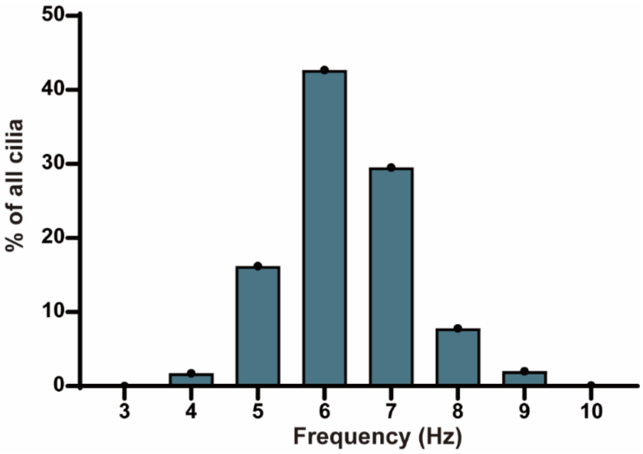

Figure S6. Calculate the frequency distribution of CBF on the screen. CBF analysis system can calculate the distribution of different frequencies.

Table S1. Summary of antibodies used in this study.

Protein/Structure/Cell	Antibody	Vendor and catalog
Tight junction	Alexa Fluor 647 anti-ZO-1	Invitrogen, Cat# MA3-39100-A647
Ciliated cell	Mouse anti-Acetyl-αTubulin	Sigma, Cat#T7451
Goblet cell	Rabbit anti-Mucin5B	Sigma, Cat#HPA008246
Secondary anitibody	Goat anti-mouse IgG, Alexa (Cy3)	Sigma, Cat# AP124C
	Goat anti-rabbit IgG, Alexa (488)	Jackson, Cat#111545003
